# Periostitis Ossificans: Report of Two Cases Resolved with Endodontic Treatment

**DOI:** 10.1155/2020/8876268

**Published:** 2020-11-24

**Authors:** Hana Bougatef, Eya Moussaoui, Ines Kallel, Mahmoud Smaoui, Lamia Oualha, Nabiha Douki

**Affiliations:** ^1^Department of Dental Medicine, EPS Sahloul, Sousse, Tunisia; ^2^Faculty of Dental Medicine, Oral Health and Oro-Facial Rehabilitation Laboratory Research (LR12ES11), University of Monastir, 5019 Monastir, Tunisia

## Abstract

Periostitis ossificans is a chronic disease characterized by an ossifying periostitis, occurring in children and young adults, commonly as a reaction to a mild infection or irritation. It is also characterized by the presence of lamellae of newly formed periosteal bone outside the cortex, giving the characteristic radiographic appearance of “onion skin.” *Aim*. The aim of this paper was to present the clinical and radiographic findings, as well as the postoperative follow-up of two cases diagnosed with periostitis ossificans of dental origin, and to discuss the differential diagnosis and treatment modalities. *Case Reports*. In the first case, a 16-year-old adolescent was referred for a persistent mandibular swelling. Intraoral examination showed two sinus tracts in relation to the carious necrotic left mandibular first molar. The periapical radiograph showed a periapical lesion in relation to the two root canals of the left mandibular first molar. Occlusal radiographs revealed the “onion skin” bone formation aspect. In the second case, a 10-year-old girl presented to our department with a slightly painful mandibular swelling. The periapical radiograph showed a periapical lesion in relation to both the mesial and distal roots of the carious necrotic right mandibular first molar. Cone beam computed tomography (CBCT) showed a subperiosteal bone formation with an “onion skin” aspect. Diagnosis of periostitis ossificans in the two cases was confirmed and the lesion was resolved by simply an endodontic treatment. *Conclusion*. Specific attention should be given to clinical and radiographic exploration in case of children with mandibular swelling. As osteosarcoma can be misdiagnosed, additional examinations, such as computed tomography, can be useful in differential diagnosis and in searching malignancy signs.

## 1. Introduction

Periostitis ossificans is a specific type of chronic sclerotic osteomyelitis, primarily affecting children and adolescents. It is characterized by a rigid bony swelling at the periphery of the jaw following a low-grade chronic odontogenic infection [1–4].

It is also known as Garre's osteomyelitis and chronic osteomyelitis with proliferative periostitis [5].

This pathological entity was first described by Carl Garré in 1893 as an irritation-inducing focal thickening of the periosteum and the cortical bone of the tibia [6].

Periostitis ossificans generally affects children and young adults. It is commonly associated with an infection of low virulence, such as odontogenic infection resulting from dental caries, mild periodontitis, dental eruption, or complication due to dental extraction [4, 7]. It affects the mandible much more commonly than the maxilla.

During the early period, a thin crust-like convex layer appears over the cortex. It is considered as a pathognomonic feature [4, 8, 9].

In this work, the clinical and radiographic findings of two cases diagnosed with periostitis ossificans of dental infection origin are presented. The differential diagnosis and treatment modalities are also discussed.

## 2. Case Reports

### 2.1. Case No. 1

A 16-year-old adolescent was referred to our department for a mandibular swelling persisting for two months. The patient had received an antibiotherapy for 7 days (Augmentin®). The patient's medical history was not contributing.

**Figure 1:**
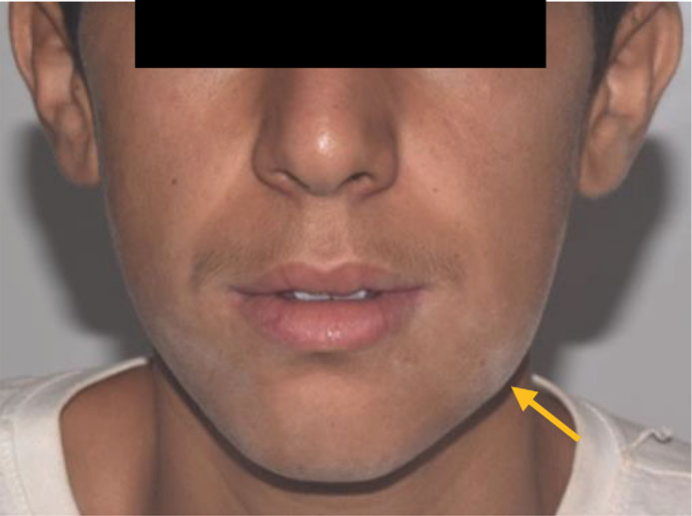
Extraoral examination revealed a left mandibular swelling.

Extraoral inspection showed facial asymmetry due to firm, nontender 1.0 × 1.5 cm mass over the lateral aspect of the left mandibular region. The overlying skin was unremarkable, and no fluctuance or discharge was noted (Figure 1).

There was no lymphadenopathy, and the patient was afebrile.

**Figure 2:**
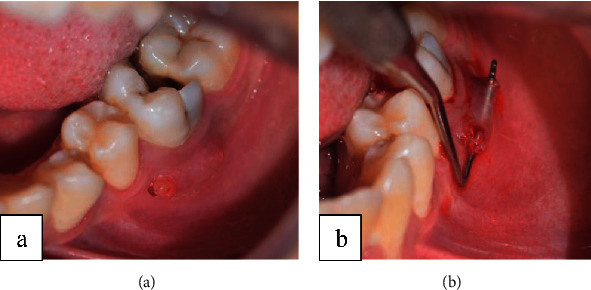
(a) A penetrating carious lesion in the first left mandibular molar. (b) Two sinus tract in relation with the first left mandibular molar.

Intraoral examination showed two sinus tracts in relation to the carious left mandibular first molar, which was painful at axial and transversal percussions, with no mobility (Figures 2(a) and 2(b)).

**Figure 3:**
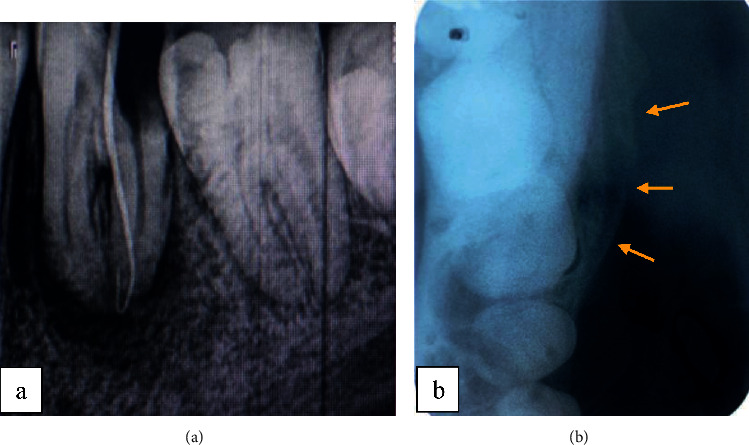
(a) Periapical radiograph revealed a not limited periapical lesion. (b) Occlusal radiograph revealed a periostitis reaction in relation with the external cortex.

The tooth was necrotic with sista 2.4 classified carious lesion. The periapical radiograph showed a periapical lesion in relation to the two root canals of the left mandibular first molar (Figure 3(a)).

The sinus tract was traced with a gutta-percha point, leading to the left mandibular first molar as shown in the periapical radiograph (Figure 3(a)).

Occlusal radiographs revealed an “onion skin” bone formation aspect (Figure 3(b)).

CBCT was not possible due to the patient's limited financial means.

Diagnosis of periostitis ossificans was confirmed by the clinical and radiographic features, as well as the causal relationship with endodontic infection.

A complete endodontic treatment was performed in only one session without further antibiotherapy.

**Figure 4:**
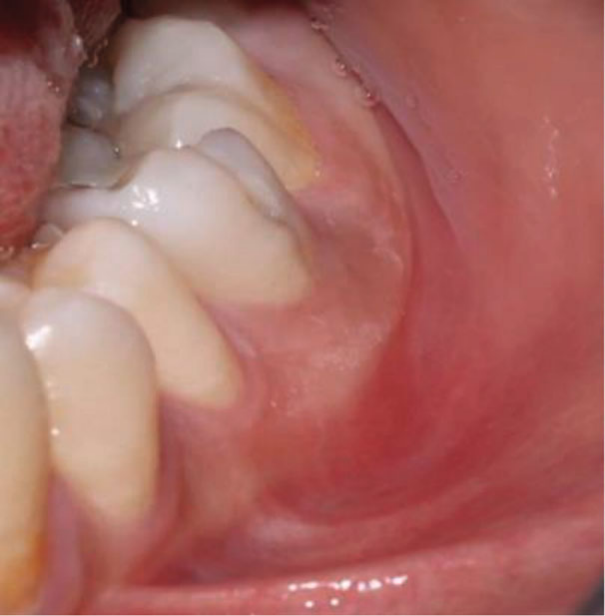
Sinus tract disappearance after 1 week.

The sinus tract disappeared in only one week (Figure 4).

**Figure 5:**
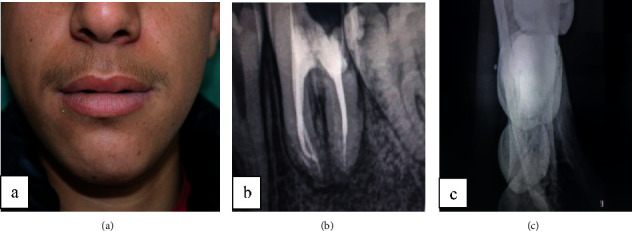
(a) Clinical healing after 4 months with extraoral swelling disappearance. (b) Periapical radiographic control after 8 months showed periapical lesion regression. (c) Occlusal radiograph showed periostitis disappearance after 4 months.

Regular follow-up, during 8 months, revealed clinical healing after 4 months with disappearance of mandibular swelling (Figure 5(a)), periapical lesion regression after 8 months (Figure 5(b)), and disappearance of periosteal reaction after 4 months (Figure 5(c)).

### 2.2. Case No. 2

A 10-year-old girl presented with a painful swelling of the right lateral and posterior mandibular region. As described by the patient's parents, swelling was progressive over five months.

Extraoral inspection showed facial asymmetry due to hard, nontender swelling in the right mandibular region.

Intraoral examination showed swelling and redness at the bottom of the vestibule in relation to the right mandibular first molar.

A temporary coronal filling was present on the tooth. It was painful at axial and transversal percussions.

**Figure 6:**
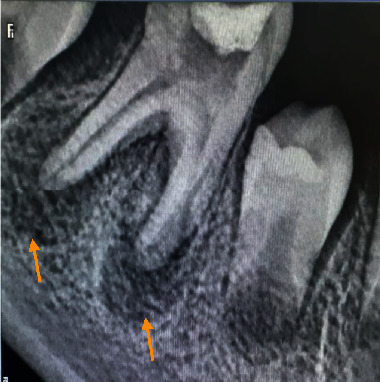
First mandibular molar with periapical lesions appended to the two root apices.

The periapical radiograph showed an open pulp chamber with temporary coronal filling and a well-limited radiolucent periapical image unilocular in relation to both the mesial and distal root of the right mandibular first molar (Figure 6).

**Figure 7:**
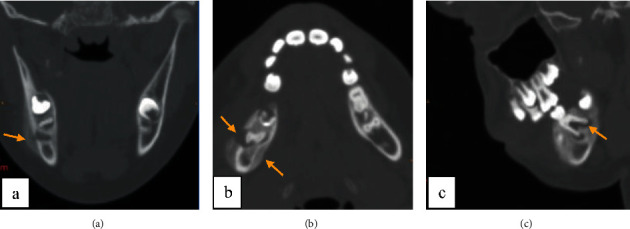
Computed tomography exam: (a) Coronal section showing “onion skin” aspect of lateral periostitis and the radiolucency in relation with first molar's roots. (b) Axial section showing periostitis in both external and internal mandibular cortex with lateral rupture of the cortical. (c) Sagittal section showing radiolucency in relation with first molar's roots.

The axial section in CBCT examination revealed the presence of about 3 lamellae of newly formed periosteal bone in the lateral and medial cortices, giving the characteristic of an “onion skin” appearance with cortical rupture in relation to the distal root (Figures 7(a)–7(c)).

**Figure 8:**
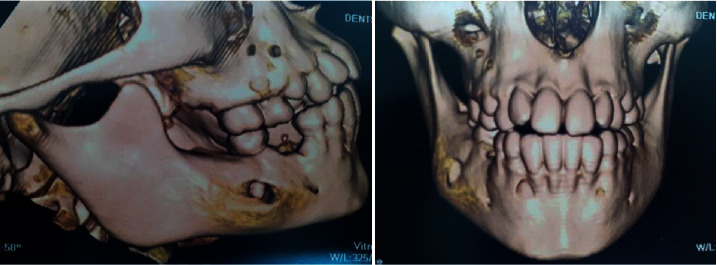
3D reconstruction showing the bone formation associated with cortical's rupture.

This was also confirmed by 3D reconstruction (Figure 8). This pathognomonic sign confirmed the diagnosis of periostitis ossificans associated with an odontogenic infection of the right mandibular first molar.

**Figure 9:**
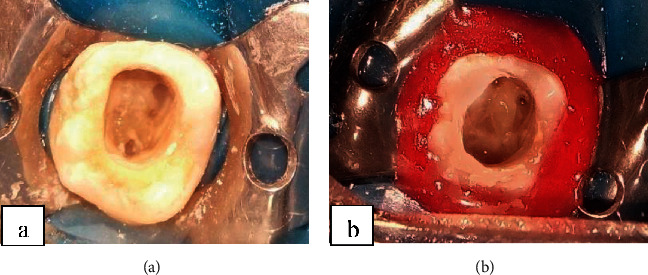
(a) Pulp stone in the chamber obliterating canal orifices. (b) Widen the canal orifices with ultrasonic tips.

In the first appointment, a pulp stone was found in the access cavity center, arguing in favor of a chronic and slow irritative phenomenon: silent necrosis. In fact, the patient did not report any pain history (Figure 9(a)). It was eliminated with ultrasonic tips (Figure 9(b)).

Root canal shaping with abundant sodium hypochlorite irrigation was carried out and endodontic medication using calcium hydroxide was placed for two weeks.

In the second and last appointment, root canal filling with gutta percha and zinc oxide eugenol as an endodontic sealer using the lateral condensation method was carried out.

The treatment was followed by clinical and radiographic monitoring after 2, 4, 8, and 12 months.

**Figure 10:**
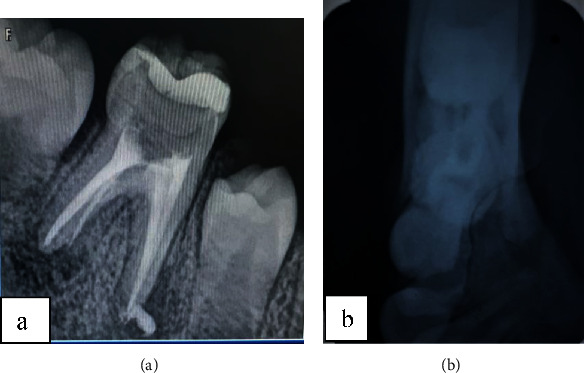
(a) Postoperative periapical radiograph after 4 months with signs of bone healing. (b) Occlusal radiograph after 4 months: partial disappearance of the periostitis.

At the 4-month follow-up, the periapical radiograph showed signs of bone healing in the periapical region (Figure 10(a)).

The occlusal radiograph showed partial disappearance of external and internal bone formation (Figure 10(b)).

**Figure 11:**
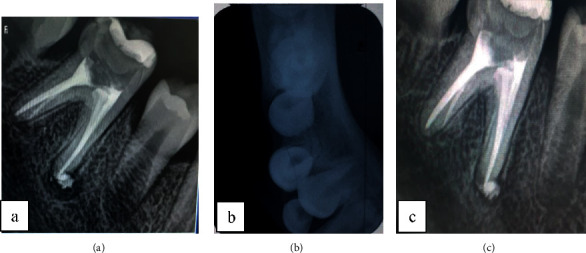
(a) Reduction of the periapical lesion size on the periapical radiograph after 8 months. (b) Occlusal radiograph showed total resolution of the periostitis after 8 months. (c) Periapical radiograph after 12 months: total regression of the apical radiolucency.

At the 8-month follow-up, the periapical radiograph showed a reduction of more than half of the primary periapical lesion size (Figure 11(a)). The occlusal radiograph revealed total healing of the periostal reaction (Figure 11(b)).

Complete bone healing was seen in the periapical radiographs with total disappearance of periapical radiolucency at the 12-month follow-up (Figure 11(c)).

**Figure 12:**
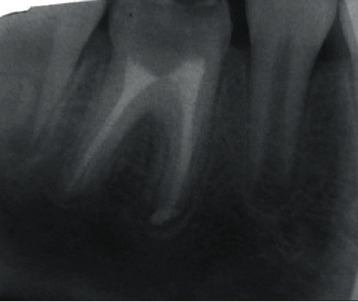
Periapical radiograph after 20 months: total regression of the apical radiolucency.

After 20 months, complete bone healing was still observed in the periapical radiograph, and the tooth was asymptomatic and functional (Figure 12).

## 3. Discussion

Periostitis ossificans refers to the diagnosis of periostitis with subperiosteal bone formation. It is also known as proliferative periostitis, characterized by reactive bone expansion as a result of rigid bony swelling at the periphery of the jaw following a chronic low-grade infection [1, 10].

It commonly occurs in children and young adults when the osteoblastic activity of the periosteum is at its peak [10]. It more often affects the mandible than the maxilla [4].

### 3.1. Ethiopathogeny and Diagnosis

In our cases, the causative agent was a pulp necrosis of the lower first molar associated with a chronic periapical infection in the two young patients.

It may be speculated that the following steps took place. A carious lesion in the mandibular first molar infected the pulp, and then it progressed to the periapical region. It extended through the cancellous bone and then through the cortical bone on the lateral aspect of the mandible. Later, the inflammatory process spread and applied pressure to the periosteum, being irritated by noxious stimuli. The periosteal osteoblasts were stimulated to form the initial bone. With episodic stimuli, bone formation continued in the form of successive layers of new bone [1, 11].

The swelling size may vary from 1 to 2 cm to reach the entire length of the jaw on the affected side. The thickness of the newly formed bone can reach 2-3 cm [7].

Gradually, the cortex thickens as a result of successive new bone deposits. This lamellar structure is referred to as an “onion skin” aspect on radiographs [4, 8, 9].

The number of laminations varies from 1 to 12. Radiolucent separation is present between the new bone and the original cortex. Within the new bone, areas of small sequestra or osteolytic radiolucencies may also be found [3, 12].

The patient's medical history usually reveals an episodic pain with dormant periods and progressive swelling. Those may be the only symptoms, but subjective signs may be variable.

The degree and duration of the symptoms depend on various factors, such as the virulence of the causative organisms, the presence of underlying diseases, and the immune status of the host [13].

This progressive evolution reveals the benign nature of this pathology, being unlike the malignant one, often characterized with a fast evolution of symptoms [14].

In our cases, the two patients presented a history of intermittent dental pain. The first patient was referred for persistent mandibular swelling, evolving for two months. The second one presented with a localized swelling, evolving for five months, according to his parents.

Clinically, this is usually manifested by facial asymmetry caused by localized unilateral mandibular hard swelling, which can be seen and it is palpable both in the extraoral and intraoral examination.

The overlying skin appears normal. Trismus and lymphadenopathy can be observed [15].

Attention should be given to the objective symptoms that are not associated with signs of malignancy, such as dental mobility, hypoesthesia, dental displacement, and severe trismus [14].

At the clinical examination, our patients presented with a small and well-limited mandibular swelling in the posterior region with neither trismus nor lymphadenopathy. The intraoral examination revealed hard swelling in the bottom of the vestibule related to the first mandibular molar. The mucosa was erythematous.

In the first case, two sinus tracts were present. The left mandibular first molar presented a carious lesion sista 2.4 and was painful at axial and transversal percussions. In the second case, the right mandibular first molar presented an incomplete endodontic treatment with occlusal temporary coronal filling.

The patients' age as well as the clinical aspect led to the diagnosis of periostitis ossificans. Ewing sarcoma and osteosarcoma, occurring at the same age, were also possible diagnoses. So, radiographic examinations were necessary to confirm the right diagnosis.

Periapical radiograph can confirm a dental low-grade infection with the presence of apical radiolucency, bone loss, marginal cyst, or extraction site.

Occlusal radiograph can explore the horizontal dimension of the jaws. Thus, if well-performed, it shows the thickened bone with lateral and/or internal periostal reaction [15].

In fact, occlusal radiographs are necessary and they may aid in establishing a diagnosis. In case of periostitis ossificans, this radiograph clearly shows the periostal reaction, characterized by a number of lamellae blowing the external jaw cortical. This is a pathognomonic radiological feature, known as the “onion skin” aspect.

However, 3D radiographic examinations is highly indicated to explore the local extension of the disease, its relationship to the anatomic structures, and its exact characteristic like density, limits, and size.

Maxillary CT scan can distinguish between the two types of periostitis: the “onion skin” aspect as a sign of benignity and the “sun ray” aspect as a sign of malignancy [16].

CBCT, being less expensive with lower dose of radiation, is also useful to show the “onion skin” aspect of the periosteal reaction on the axial and coronal sections [16].

In the first case, preoperative radiographs showed a deep carious lesion penetrating in the pulp chamber in relation to the left mandibular first molar with a well-limited radiolucent periapical image unilocular in relation to both the mesial and distal tooth root.

Due to the patient's limited financial conditions, only an occlusal radiograph was performed to confirm our diagnosis, and it showed the “onion skin” aspect of the periosteal reaction.

In the second case, preoperative radiographs showed an opened access cavity having temporary coronal filling with a periapical lesion.

As the patient had already performed a CBCT imaging, occlusal radiographs were only useful for follow-up and monitoring. They were used to see the external cortical aspect, the regression of bone formation, the healing sign, and the favorable evolution.

In the CT scan, the axial and coronal sections showed a localized bone formation with an “onion skin” aspect, a sign of benignity, and a pathognomonic feature of periostitis ossificans.

### 3.2. Differential Diagnosis

Periostitis ossificans should be distinguished from benign and malignant pathologies causing bone formation. Most of them usually develop in the same age range [4, 8, 17].

Ewing's sarcoma and osteosarcoma occur in the same age presenting with hard swelling, similar to periostitis ossificans. However, they can be distinguished from periostitis ossificans by the clinical complications (dental mobility, facial neuralgia, and lip paresthesia) and the radiological “sun ray” aspect, causing very rapid bone enlargement and more osteolytic reactions in the bone [2, 18].

Fibrous dysplasia should have been included in the differential diagnosis. It is classified as bone-related lesions [19].

The symptoms of fibrous dysplasia and periostitis ossificans may be clinically indistinguishable [8, 20].

It can be seen at an early age, and the bone proliferation is similar to periostitis in both shape and volume. However, fibrous dysplasia is distinguished by the radiographic aspect of “ground glass appearance” as well as the cortex thinning [2, 4, 8].

Caffey disease is a genetic bone disorder that could have the same appearance of an “onion skin” as periostitis ossificans. However, it is distinguished from periostitis ossificans by the early age of onset (prior to two years of age). It is more common in the ramus and the mandiblar angulus region with bilateral involvement and occurrence in multiple bones [2].

Primary chronic osteomyelitis (PCO) is an uncommon nonbacterial chronic inflammatory disease of unknown etiology. It can be associated with other conditions, such as autoimmune diseases and syndromes, including Majeed syndrome, cherubism, and “SAPHO (Synovitis, Acne, Pustulosis, Hyperostosis, and Osteitis) syndrome” [21].

PCO of the jaw has been reported in children. The mandible is the most affected site in the maxillofacial region. Clinically, it is characterized by acute pain and aseptic swelling.

Radiological examination reveals the presence of radiolucent areas combined with progressive osteosclerosis and laminations of the periosteum, without the presence of a dental origin [22].

Primary tuberculous osteomyelitis is a rare form of osteomyelitis caused by tuberculosis. In children, the mandibular region is very unlikely to have higher frequency compared to the maxilla.

Clinically, it is characterized by pain, swelling, and discharge through the intra or extraoral sinus tract. Radiographically, it appears as blur radiolucency with erosion of the cortex. The bone is gradually replaced by granulomatous tissue.

Pathologically, diagnosis can be confirmed by the presence of tuberculous granulomas [23, 24].

In our cases, mild odontogenic infection was a proven cause. The clinical examination (slow evolution, absence of dental mobility, and hypoesthesia) and the radiographic aspects, especially the “onion skin” aspect, were sufficient to establish the diagnosis. It was then confirmed by the favorable evolution following treatment.

Often, clinical and radiologic findings are sufficient to establish diagnosis. Yet, histological examinations are sometimes necessary to confirm diagnosis [25–27].

When performed, they reveal a benign fibro-osseous tissue with peripheral osteoblastic activity due to a reactive new bone deposition. New periosteal deposits are parallel to each other with respect to the bone cortex.

Central osteoblasts and osteoclasts are evident. Lymphocytes and plasma cells are found in the marrow space [15].

### 3.3. Treatment and Evolution

There has always been a dilemma in planning the treatment strategies for periostitis ossificans because of some update in the classification of mandibular osteomyelitis [4].

Periostitis ossificans has long been considered like other types of osteitis that can arise from either local extension or hematogenous spread. For this reason, the treatment plan has been radical for many years, and it has been based on extraction with long term antibiotherapy [28].

With the evolution of diagnostic terms over the years, different opinions appeared regarding the most suitable treatment plan for periostitis ossificans [9].

The treatment plan can be conservative or radical, with or without associated antibiotic therapy [9].

The role of endodontic therapy in the management of periostitis ossificans has been questionable. Batcheldor et al. suggested the possible efficacy of tooth conservation and endodontic intervention [8, 29].

Tooth conservation with an early adequate root canal treatment with or without antibiotherapy has been highlighted in the last few years. In fact, it was proven through some reported cases that endodontic treatment alone is sufficient and able to resolve this pathology.

Even more, hyperbaric oxygen therapy and endodontic treatment have proven to be successful [1, 30, 31].

In the present case reports, endodontic treatment was the treatment of choice due to the patients' young age, the possibility of maintaining the tooth, and the positive attitude of the patients and their parents toward this treatment plan.

The primary challenges of root canal treatment are to eliminate infection, secure healing of the bone lesion, and therefore conserve the tooth [32–34]. Indeed, optimal prognosis depends on the ability to create favorable conditions for adequate healing.

To improve the success rate of endodontic treatment, especially for necrotic teeth, more focus should be put on the chemo-mechanical preparation.

In fact, disinfection of the root canal space is the key to eradicate bacteria within the root canal, leading to periapical healing. This is ensured by both root canal shaping and irrigation [35].

Because of its complexity, the root canal system of a necrotic tooth harbors thousands of microorganisms. They are found especially within a biofilm form, adhering firmly to the dentinal walls and resisting to the irrigation solutions [35].

The strategies recommended for improving disinfection after chemo-mechanical preparation include the use of an interappointment endodontic medication or an optimized one-visit endodontic treatment [36].

Many studies, using microbial culture, have confirmed the advantage of using endodontic medication based on calcium hydroxide to complete root canal disinfection [37].

However, many other studies have proven that the 2-visit protocol involving an interappointment medication together with calcium hydroxide results in an improved microbiological status of the root canal system when compared to the 1-visit protocol. They suggested that residual surviving microorganisms are entombed by root space obturation, and they die due to the absence of nutrients [37].

Yet, this topic is still a subject of debate and some studies have shown controversial conclusions.

In the first case, endodontic treatment was performed in one visit. Root canal filling was carried out using bioceramic sealers for their antibacterial proprieties. Fortunately, the result was satisfying with periapical healing.

In the second case, as the patient did not support long sessions, root canal dressing was placed based on calcium hydroxide during two weeks. This was intended to enhance disinfection.

Concerning the antibiotherapy, according to the literature, there is no consensus about its use as well as the treatment duration, varying from six, eight weeks to twelve months [38].

In our cases, the patients received antibiotherapy (Augmentin®) for seven days. It was given by the child's parents in the first case and by the dentist in the second case. The prescription was not renewed.

The efficacy of nonsteroidal anti-inflammatory drugs (NSAIDs) with regard to Garré's sclerosing osteomyelitis of the jaw bone is not clear. Few reports have focused on this treatment strategy because most of the cases were resolved with certain dental managements [39].

For better management, regular follow-up is crucial until complete healing is achieved [1].

In fact, when endodontic treatment is performed, long-term follow-up should be conducted. Biopsy is indicated if the lesion continues to increase in size after an apparently successful treatment [8, 29].

For lesions of limited size, we usually obtain good therapeutic effects and favorable prognosis within few months.

After the elimination of the infection source, the bone gradually remodels itself and the original facial symmetry will be restored. This bone remodeling may be improved by the overlying muscle pull, which is attached to it [9].

In the two cases reported, our therapeutic attitude was successful. At the 12-month follow-up, bone healing was noted without any other complications.

The first patient was followed-up for 11 months. Periosteal reaction healing was observed at 4 months with mandibular swelling disappearance. Then, complete periapical healing was seen at 11 months.

The second patient was followed-up for 20 months. Periosteal reaction healing was observed at 4 months. Regression of periapical radiolucency was seen at 12 months.

Thus, for the two cases, there was no need for surgery as response to conventional endodontic treatment was favorable.

It is known that periostitis ossificans is curable if adequate treatment is given. However, if the correct diagnosis is delayed by more than 6 months, it may progress into a persistent and deforming form [9].

Surgical remodeling can be performed if no spontaneous regression is present, especially when adequate diagnosis and management are perfectly achieved [8].

## 4. Conclusion

Periostitis ossificans is a pathology coexisting with some conditions, including chronic infection in young patients with an energetic osteoblastic activity in the periostum and an equilibrium between the virulence of the causal agent and the host resistance.

Specific attention should be given to the clinical and radiographic explorations in case of mandibular swelling in order not to miss a possible malignant bone disease.

The treatment goal of periostitis ossificans is to eradicate the source of infection. Whenever the clinical situation permits, endodontic therapy should be the main treatment choice. Antibiotherapy is not systemic.

In our reported cases, endodontic treatment was the approach of choice and it led to a favorable evolution with complete regression.

## References

[1] Brazão-Silva M. T., Pinheiro T. N. (2017). The so-called garrè's osteomyelitis of jaws and the pivotal utility of computed tomography scan. *Contemp Clin Dent*.

[2] Karjodkar F. R. (2009). *Textbook of Dental and Maxillofacial Radiology*.

[3] Neville B. W., Damm D. D., Allen C. M., Bouquot J. E., Neville B. W. (1995). Pulp and periapical diseases. *Oral and Maxillofacial Pathology*.

[4] Suma R., Vinay C., Shashikanth M. C. (2007). Garre's sclerosing osteomyelitis. *Journal of the Indian Society of Pedodontics and Preventive Dentistry*.

[5] Neelima M., Neelima M. (2012). Osteomyelitis and osteo radionecrosis of the jaw bones. *Textbook of Oral and Maxillofacial Surgery*.

[6] Garré C. (1893). UeberbesondreFormen und Folgezustande d. akuteninfekt. *Osteomyelitis Beitr z klin Chir*.

[7] Erişen M., Bayar OF, Ak G. (2014). Garre osteomyelitis: a case report. *J Dent Fac Atatürk Univ*.

[8] Gonçalves M., Oliveira D. P., Oya E. O., Gonçalves A. (2002). Garre’s osteomyelitis associated with a fistula: a case report. *The Journal of Clinical Pediatric Dentistry*.

[9] Kannan S. K., Sandhya G., Selvarani R. (2006). Periostitis ossificans (Garrè's osteomyelitis) radiographic study of two cases. *International Journal of Paediatric Dentistry*.

[10] Oulis C., Berdousis E., Vadiakas G., Goumenos G. (2000). Garre’s osteomyelitis of an unusual origin in a 8-year-old child. a case report. *International Journal of Paediatric Dentistry*.

[11] Cohen S., Burns R. (1980). *Pathways of the Pulp*.

[12] Liu D., Zhang J., Li T. (2019). Chronic osteomyelitis with proliferative periostitis of the mandibular body: report of a case and review of the literature. *Annals of the Royal College of Surgeons of England*.

[13] Suei Y., Taguchi A., Tanimoto K. (2005). Diagnosis and classification of mandibular osteomyelitis. *Oral Surgery, Oral Medicine, Oral Pathology, Oral Radiology, and Endodontics*.

[14] Zaghbani A., Ben Yousef S., Oualha L., Hasni W., Souid K., Baccouche C. (2011). Jaw malignancies: signs that should alert the dentist. *Tunis Med*.

[15] Belli E., Matteini C., Andreano T. (2002). Sclerosing osteomyelitis of Garré periostitis ossificans. *The Journal of Craniofacial Surgery*.

[16] Schulze D., Blessmann M., Pohlenz P., Wagner K. W., Heiland M. (2006). Diagnostic criteria for the detection of mandibular osteomyelitis using cone-beam computed tomography. *Dento Maxillo Facial Radiology*.

[17] Nakano H., Miki T., Aota K., Sumi T., Matsumoto K., Yura Y. (2008). Garré's osteomyelitis of the mandible caused by an infected wisdom tooth. *Oral Sci Int*.

[18] White S. C., Pharoah M. J. (2009). *Oral Radiology: Principles and Interpretation*.

[19] Barnes L., Eveson J. W., Reichart P., Idransky D. (2005). *Pathology and Genetics of Head and Neck Tumours*.

[20] Pitak-Arnnop P., Bellefqih S., Bertolus C. (2008). Ewing’s sarcoma of jaw bones in adult patients: 10-year experiences in a Paris university hospital. *Journal of Cranio-Maxillo-Facial Surgery*.

[21] Stern S. M., Ferguson P. J. (2013). Autoinflammatory bone diseases. *Rheumatic Disease Clinics*.

[22] Berglund C., Ekströmer K., Abtahi J. (2015). Primary chronic osteomyelitis of the jaws in children: an update on pathophysiology, radiological findings, treatment strategies, and prospective analysis of two cases. *Case Reports in Dentistry*.

[23] Kalaiarasi R., Vijayakumar C., Archana R., Natarajan R. (2018). Pediatric primary tuberculous osteomyelitis of the mandible mimicking parotitis. *Cureus*.

[24] Natarajarathinam G., Rao A. V., Palanimuthu S., Kannaperuman J. (2013). Primary tuberculous osteomyelitis of the mandible: a rare case report. *Dental Research Journal*.

[25] Benca P. G., Mostofi R., Kuo P. C. (1987). Proliferative periostitis (Garré's osteomyelitis). *Oral Surgery, Oral Medicine, and Oral Pathology*.

[26] Felsberg G. J., Gore R. L., Schweitzer M. E., Jui V. (1990). Sclerosing osteomyelitis of Garrè (periostitis ossificans). *Oral Surgery, Oral Medicine, and Oral Pathology*.

[27] Martin-Granizo R., Garcia-Gonzalez D., Sastre J., Diaz F. J. (2016). Mandibular sclerosing osteomyelitis of Garré. *Otolaryngology–Head and Neck Surgery*.

[28] Krakowiak P. A. (2011). Alveolar osteitis and osteomyelitis of the jaws. *Oral and Maxillofacial Surgery Clinics of North America*.

[29] Batcheidor G. D., Giansanti J. S., Hibbard E. D., Waldron C. A. (1973). Garré’s Osteomyelitis of the Jaws: A Review and Report of Two Cases. *Journal of the American Dental Association (1939)*.

[30] Akgül H. M., Çağlayan F., Günen Yılmaz S., Derindağ G. (2018). Garre's osteomyelitis of the mandible caused by infected tooth. *Case Reports in Dentistry*.

[31] Jayasenthil A., Aparna P. V., Balagopal S. (2015). Non-surgical endodontic management of Garre's osteomyelitis: a case report. *British Journal of Medicine and Medical Research*.

[32] Jacobson H. L. J., Baumgartner J. C., Marshall J. G., Beeler W. J. (2002). Proliferative periostitis of Garré: report of a case. *Oral Surgery, Oral Medicine, Oral Pathology, Oral Radiology, and Endodontics*.

[33] Mattison G. D., Gould A. R., George D. I., Neb J. L. (1981). Garre’s osteomyelitis of the mandible: the role of endodontic therapy in patient management. *Journal of Endodontia*.

[34] McWalter G. M., Schaberg S. J. (1984). Garre’s osteomyelitis of the mandible resolved by endodontic treatment. *Journal of the American Dental Association (1939)*.

[35] Eliyas S., Briggs P. F., Porter R. W. (2010). Antimicrobial irrigants in endodontic therapy: 1. Root canal disinfection. *Dental Update*.

[36] Siqueira J. F., Rôças I. N. (2011). Optimising single-visit disinfection with supplementary approaches: a quest for predictability. *Australian Endodontic Journal*.

[37] Vera J., Siqueira J. F., Ricucci D. (2012). One- versus two-visit endodontic treatment of teeth with apical periodontitis: a histobacteriologic study. *Journal of Endodontia*.

[38] Bah A., Camara S., Vaysse F., Bailleul-Forestier I. (2016). Prise en charge de l'Ostéopériostite chronique dentaire chez l'enfant: A propos d'un cas. *Afr J Dent Implantol*.

[39] Schultz C., Holterhus P. M., Seidel A. (1999). Chronic recurrent multifocal osteomyelitis in children. *The Pediatric Infectious Disease Journal*.

